# Burden of Care in Caregivers of Iranian patients with chronic disorders: a systematic review and meta-analysis

**DOI:** 10.1186/s12955-020-01503-z

**Published:** 2020-08-03

**Authors:** Hayedeh Rezaei, Seyed Hassan Niksima, Reza Ghanei Gheshlagh

**Affiliations:** 1 Department of Nursing. Faculty of Nursing and Midwifery, Kurdistan University of Medical Sciences, Sanandaj, Iran; 2 Preventive Medicine and Public Health Research Center, Iran University of Medical Sciences, Tehran, Iran; 3 Spiritual Health Research Center, Research Institute for Health Development, Kurdistan University of Medical Sciences, Sanandaj, Iran

**Keywords:** Burden of care, Caregiver, Chronic disorder, Systematic review, Iran

## Abstract

**Introduction:**

Caring for patients with chronic disorders can lead to different problems for caregivers in physical, psychological, social, family, and financial domains. High levels of burden of care can make caregivers vulnerable to physical and psychological conditions and influence their quality of life. Therefore, the goal of the present study was to estimate the overall percentage of burden of care in caregivers of Iranian patients with chronic disorders.

**Methods:**

A total of 25 articles published from inception to February 2019 were reviewed. Search for articles was conducted in international (Scopus, Web of Science, and PubMed) and domestic (Scientific Information Database (SID) and MagIran) databases, using the following keywords: “Caregiver,” “Burden,” and “Iran,” and their possible combinations. The data were analyzed using the meta-analysis method and the random effects model. All the analyses were performed using STATA, version 14.

**Results:**

The overall percentage of burden of care in caregivers of Iranian patients with chronic disorders was 53.28% (95% CI: 46.13–60.43). The highest percentage of burden of care was related to dialysis (62.75; 95% CI: 56.11–69.38), mental disorders (58.69; 95% CI: 49.70–67.69), and Alzheimer’s disease (57.07; 95% CI: 46.23–67.92), respectively; and the lowest percentage of burden of care was related to diabetes (34.92; 95% CI: 18.01–51.82).

**Conclusions:**

Caregivers of Iranian patients with chronic disorders experience high levels of burden of care, especially those caring for patients undergoing dialysis, patients with mental disorders, and patients with Alzheimer’s disease. Therefore, necessary measures need to be taken by Iranian health care officials to reduce burden of care in caregivers.

## Introduction

Chronic disorders are the main cause of mortality around the world, so that they account for 60% of deaths globally [1]. Chronic disorders threaten and alter patients’ well-being; independence; body integrity; family, social, and professional roles; personal goals and plans for the future; and economic stability [2]. In most cases, it is family members who take care of patients [3], therefore, the treatment of chronic disorders and taking care of chronic patients has transferred into homes [4]. Taking care of a patient can affect all aspects of the caregiver’s life and lead to multiple mental, emotional, physical, and financial challenges for them [5]. In addition, caregiving can lead to social isolation, life dissatisfaction, reduced quality of life, and reduced physical health in caregivers [6–9], and make them vulnerable to depression, anxiety, and stress [10]. The more demands placed on the caregiver by the patient, the more challenges the caregiver experiences [11]. In addition to mental problems, caregiving can lead to physical problems, including gastric ulcers, back injuries, headaches, arthritis, and high blood pressure [12]. Therefore, caregivers are sometimes called hidden patients [13]. A previous study found higher mortality rates (by 63%) in caregivers experiencing caregiver strain than other caregivers [14].

Burden of care refers to physical, mental, social, or financial reactions by the caregiver during caregiving shown as a result of an imbalance between patient’s needs and health services [15]. This lack of balance is related to caregivers’ multiple roles, physical and mental condition, and financial status; and quality of governmental health services. It also has the following consequences: impairment in daily and leisure activities and social interactions, disablement and illness, isolation from family and losing family relationships, losing hope in social support, inadequate care received by the patient, vulnerability to chronic disorders, and abandoning the patient [16]. Caregivers have to maintain a balance between their own needs and those of their patients. They experience a high level of stress due to lack of adequate training on caregiving and limited resources [17, 18*, 19*]. On the other hand, because they spend most of their time with patients, they tend to forget their own needs and this may alter their lifestyle [20, 21*, 22].

Goldzweig et al. showed that caregivers of cancer patients experienced high levels of burden of care and low levels of social support and lacked the skill to adjust to high levels of burden of care [23]. Burden of care is directly related to patients’ needs [13]. Another study found that caregivers with lower education and income levels reported higher levels of burden of care [24]. In Iran, like other developing countries, due to an imbalance between the number of patients and that of health care providers, caregivers are responsible to take care of their patients, and this imposes high levels of physical, mental, social, emotional, and financial burden on them; determining the level of burden of care can highlight this issue properly. Therefore, the goal of the present study is to estimate the percentage of burden of care in caregivers of chronic patients in Iran.

## Methods

In this systematic review and meta-analysis, the percentage of burden of care in caregivers of Iranian patients with chronic disorders was reviewed and reported according to the steps of the PRISMA statement [25]. Based on the PICO, Population (P) includes articles focused on burden of care in caregivers of Iranian patients with chronic disorders, and Outcome (O) is the burden of care raw score. Intervention (I) and Comparison (C) are not applicable.

### Search strategy

To find related articles, two researchers independently searched the following national and international scientific databases until February 2019: SID, MagIran, Google Scholar, Web of Science, PubMed, and Scopus. Search for articles, screening the articles, methodological quality examination, and data extraction were all conducted by two independent researchers, and any disagreement between them was resolved by a third author experienced in this matter.

The reference lists of articles were also reviewed in order to find more related articles. Keywords of “burden,” “Caregiver,” “Iran,” and their possible combinations were used to search for articles. In the first step, all articles containing the above keywords were collected. In order not miss any article names of chronic disorders (e.g. diabetes, stroke, heart disease, Alzheimer’s disease, mental disorders, cancer, social cord injury, thalassemia etc.) were used along with the aforementioned keywords.

### Selection of studies and data extraction

Articles with the following criteria were included in the study: observational (non-interventional) studies, published in Farsi or English, providing enough information related to the study objectives, referring to name of a chronic disorder in title, and reporting the burden of care raw score. On the other hand, articles with the following criteria were excluded from the study: lacking sufficient information, not presenting new results (repeated studies), and unavailable full texts. According to the aforementioned criteria, abstracts were reviewed by the researchers and the related ones were selected. The data was extracted and managed in a pre-designed form in Microsoft Excel. Then, a form assessing name of the first author, publication year, study participants, sample size, measuring instruments, location of research, and burden of care standardized score was used to assess article characteristics.

### Transformation of scale scores

In the next step, each raw score was transferred to a 0–100 scale using the following formula:

$$ \mathrm{Transformed}\ \mathrm{Scale}=\left[\frac{\left(\mathrm{Actual}\ \mathrm{raw}\ \mathrm{score}\right)-\left(\mathrm{lowest}\ \mathrm{possible}\ \mathrm{raw}\ \mathrm{score}\right)}{\mathrm{possible}\ \mathrm{raw}\ \mathrm{score}\ \mathrm{range}}\right]\times 100 $$

In the formula shown above, “Actual raw score” is the raw values obtained by summation, “lowest possible raw score” is the lowest raw value possible, and “possible raw score range” is the difference between the maximum and minimum possible raw scores [26].

The three following instruments were used to assess burden of care in the selected studies:

The Caregiver Burden Inventory (CBI): This 24-item questionnaire was designed by Novak and Guest in 1989 to assess objective and subjective burden of care. It has 5 subscales, including physical, developmental, emotional, social, and time-dependence burden. Items are rated on a 5-point Likert-type scale ranging from 1 (totally incorrect) to 5 (totally correct). Total score ranges from 24 to 120, and higher scores indicate higher burden of care [27].

The Zarit Caregiver Burden Interview (ZBI): This interview was developed by Zarit et al. It has 22 items assessing personal, social, emotional, and financial pressures. The items are rated on a 5-point Likert-type scale ranging from 0 (always) to 4 (nearly always). Total score rages from 0 to 88, and higher scores show greater burden of care [28].

The Modified Caregiver Strain Index (MCSI): This was developed by Mohammadi (2006) based on the index developed by Robinson. It has 13 items assessing burden of care in caregivers of patients with Alzheimer’s disease. The items are rated on a 4-point Likert-type scale ranging from 1 (little) to 4 (very much). Total score ranges from 13 to 52, and higher scores indicate greater burden of care [29*].

### Quality assessment

Methodological quality of the papers was investigated based on the ten selected items from the STROBE (Strengthening The Reporting of OBservational Studies in Epidemiology) checklist (title and abstract, objectives and hypotheses, research context, entry criteria, sample size, statistical methods, descriptive data, interpretation of findings, research limitations and funding) [30]. Based on the methodological quality score, articles are categorized as follows: weak (score 0–4), average (score 5–7), and strong (score 8–10). Articles with low methodological quality were excluded from the analysis. More information on methodological quality is provided in Table 1.

**Table 1:** The methodological quality based on STROBE

First Author	Title & abstract	Objectives and hypotheses	Research setting	Inclusion criteria	Sample size	Statistical methods	Descriptive data	Analysis of findings	Limitations	Funding	Score
Asadi [31*]	+	+	+	+	+	+	+	+	_	+	9
Azimi Lolaty [32*]	+	+	+	+	+	+	+	+	_	_	8
Adili [33*]	+	+	+	+	+	+	+	+	_	_	8
Jafari [34*]	+	+	+	+	+	+	+	+	+	+	10
Hassanzadeh [35*]	+	+	+	+	+	+	_	+	+	+	9
Rahimi Naderi [36*]	+	+	+	+	+	+	+	+	_	+	9
Mirsoleimani [37*]	+	+	+	+	+	+	+	+	_	+	9
Torabi Chafjiri [38*]	+	+	+	+	+	+	+	+	_	+	9
Safaeian [39*]	+	+	+	+	+	+	+	+	_	+	9
Khazaeipour [40*]	+	+	+	+	+	+	+	+	+	_	9
Bamari [41*]	+	+	+	+	+	+	+	+	_	_	8
Vahidi [42*]	+	+	+	+	+	+	+	+	_	_	8
Talebi [18]	+	+	+	+	+	+	+	+	_	+	9
Haghgoo [43*]	+	+	+	+	+	+	+	+	_	_	8
Hosseini [44*]	+	+	+	+	+	+	+	+	_	+	9
Mashayekhi [21]	+	+	+	_	+	+	+	+	_	+	8
Shamsaei [45*]	+	+	+	+	+	+	+	+	+	+	10
Mashayekhi [46*]	+	+	+	+	+	+	+	+	_	_	8
Garmabi [47*]	+	+	+	+	+	+	+	+	_	_	8
Salmani [19*]	+	+	+	+	+	+	+	+	+	_	9
Abbasi [48*]	+	+	+	+	+	+	+	+	+	+	10
Abdollahpour [49*]	+	_	+	+	+	+	+	+	+	_	8
Haresabadi [50*]	+	+	+	+	+	+	+	+	+	+	10
Navidian [51*]	+	+	+	+	+	+	+	+	_	_	8
Mohammadi Shahbalagy [29*]	+	+	+	+	+	_	+	+	_	_	8

### Statistical analyses

Since the burden of are score had a normal distribution, variance of each study was calculated based on the normal distribution, as $$ \mathit{\operatorname{var}}\left(\overline{X}\right)=\raisebox{1ex}{${\sigma}^2$}\!\left/ \!\raisebox{-1ex}{$n$}\right. $$. The weight of each study was inversely proportional to the variance. The burden of care mean score was evaluated with a 95% confidence interval. The I^2^ statistic and the Cochran Q test were used to assess heterogeneity among the data. For I^2^ statistics greater than 50% or Cochran Q test probability values less than 0.05 (*P* < 0.05), the random effects model was used [52]. Otherwise, the fixed effects model was used. The sensitivity analysis was used to determine the role of each study in the final result. This was done by removing the studies one at a time, and assessing the impact of removing each study on the final results. A meta-regression model was employed to assess the relation between burden of care scores and mean age of participants, year of publication, and sample size. Publication bias was inspected visually with funnel plots [53] and analyzed with Egger’s method [54]. The data was analyzed using the Stata software, version 14. The significance level was set at 0.05.

## Results

**Fig. 1:**
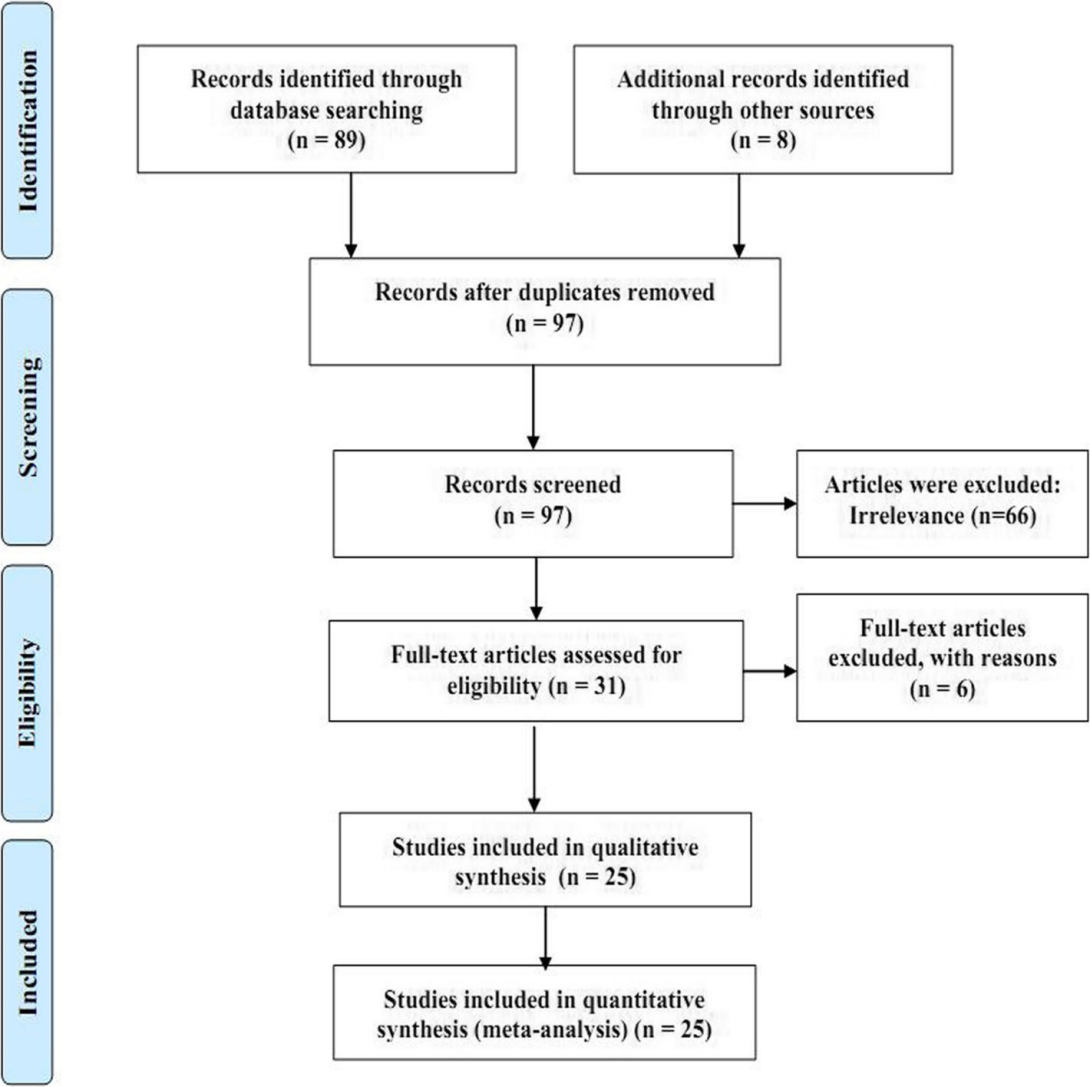
Article selection and screening flowchart

According to the first step of the PRISMA guidelines, in the stage of identification, 97 articles were retrieved from domestic and international databases. In the screening stage, abstracts were examined, and 66 articles with unrelated subjects were discarded. In the stage of eligibility examination, another 6 articles were discarded based on the inclusion and exclusion criteria. Finally, a total of 25 articles were included in the analysis. The process of searching for, selecting, and screening articles is shown in Fig. 1.

From the selected articles, 12 were in English and 13 in Farsi. More information on year of publication, type of chronic disorders, instruments to measure burden of care, burden of care standard score, methodological quality, and demographic description of participants is shown in Table 2. Quality score refers to the methodological quality of studies. This score is calculated based on the 10 aforementioned items and total methodological quality score ranges from 0 to 10. Higher scores indicate greater methodological quality.

**Table 2:** Details of selected articles

First Author	Year	Sample size	Type of patients	Scale	Burden of care (%)	Subject Description
Asadi [31*]	2018	152	Psychology	ZARIT	35.21	Gender of the caregivers: 98 men and 54 women 50% of the caregivers were 30–50 years old. 97% of the caregivers were married. 60.6% of the caregivers had a high school diploma or a lower education level.
Azimi Lolaty [32*]	2018	200	Psychology	ZARIT	64.77	Gender of the caregivers: 104 men and 96 men Mean age of the caregivers: 51.2 ± 12.9 years 57.4% of the caregivers were 40–59 years old. 48.5% of the caregivers were children of patients.
Adili [33*]	2018	116	Cancer	CBI	18.25	Mean age of the patients: 46 ± 11 years Mean age of the caregivers: 48 ± 10 years 85% of the caregivers were wives of husbands of patients. 57% of the caregivers had a high school diploma or a lower education level.
Jafari [34*]	2018	246	Dialysis	CBI	67.5	Gender of the patients: (129 men and 116 women) Mean age of the patients: 58.6 ± 15 years Duration of receiving hemodialysis: 4.12 ± 3.74 years Gender of the caregivers: 146 (165 women and 81 men) Mean age of the caregivers: 42 ± 15 years Marital status, job, and education of the caregivers: 153 (62.2%) married, 133 (53.9%) housewives, and 118 (48.1%) with a high school diploma
Hassanzadeh [35*]	2017	200	Psychological problems	CBI	52.86	Gender of the patients: 91 women and 109 men Most of the patients (43%) were 11–15 years old. Gender of the caregivers: 87 men and 113 women
Rahimi Naderi [36*]	2017	129	Diabetes	CBI	26.85	Mean age of the caregivers: 39 ± 12 years Gender of the caregivers: 101 women and 28 men 31.8% of the caregivers were daughters of patients. 77.5% of the caregivers were married.
Mirsoleimani [37*]	2017	104	Cancer	CBI	38.45	Gender of the patients: (33 men and 71 women) Gender of the caregivers: (46 men and 58 women) 64 (61.5%) the patients had breast cancer. 50 (48.1%) the caregivers were children of patients. Most of the caregivers (50%) were 25–44 years old, and most of the patients (47.1%) were 45–64 years old.
Torabi Chafjiri [38*]	2017	407	Stroke	CBI	29.16	Gender of the caregivers: 362 women and 45 men Mean age of the caregivers: 38.3 ± 8.8 years Mean duration of caregiving: 4.2 ± 2.5 years Most of the caregivers (65.6%) were housewives. 96.6% of the caregivers had a high school diploma or a lower education level.
Safaeian [39*]	2017	100	Cancer	CBI	77.56	Gender of the patients: 55 women and 45 men Mean age of the caregivers: 38.1 ± 12.5 years Gender of the caregivers: 58 women and 42 men 64% of the caregivers were children of patients. 43% of the caregivers had primary school education.
Khazaeipour [40*]	2017	163	SCI	ZARIT	44.20	Mean age of the patients: 36 ± 12.5 years Gender of the patients: 32 women and 131 men Duration of suffering from the illness: 76.5 ± 79 months Marital status of the patients: 56.5% married, 38% single, and 5.5% divorced Mean age of the caregivers: 38.1 ± 13.2 years Duration of caregiving: 69.4 ± 73.1 months Gender of the caregivers: 61 men and 102 women
Bamari [41*]	2016	70	Diabetes	ZARIT	44.14	Gender of the patients: 40 women and 30 men Gender of the caregivers: 36 women and 34 men 70% of the caregivers were wives or husbands of patients.
Vahidi [42*]	2016	150	Cancer	ZARIT	34.71	Gender of the caregivers: 73 women and 77 men Mean age of the caregivers: 39.6 ± 13.8 years 34.7% of the caregivers were wives or husbands of patients.
Talebi [18]	2016	154	Dialysis	ZARIT	57.67	Mean age of the patients: 60.9 years Mean age of the caregivers: 43.7 years Duration of receiving hemodialysis: 43.2 months Most of the caregivers had a high school diploma or a lower education level (51.3%) and were married (82.5%). 49.4% of the caregivers were children of patients.
Haghgoo [43*]	2016	246	Psychology	CBI	74.19	Mean age of the caregivers: 34.5 ± 13.7 years Gender of the caregivers: 115 men and 131 women Marital status of the caregivers: 107 single, 127 married, and 12 divorced.
Hosseini [44*]	2015	150	Alzheimer’s disease	MCSI	61.97	Mean age of the caregivers: 46.7 ± 10 years 75.3% of the caregivers were married and 65.3% had a high school diploma. 66% of caregivers were daughters of patients
Mashayekhi [21]	2015	51	Dialysis	ZARIT	61.3	Mean age of the patients: 53 ± 17.9 years Gender of the patients: 22 women and 29 men Mean age of the caregivers: 42.1 ± 14.7 years Gender of the caregivers: 35 women and 16 men Most of the caregivers were married (86.3%) and illiterate (51%).
Shamsaei [45*]	2015	225	Psychology	ZARIT	58.78	Number of patients: 121 men and 104 women Number of caregivers: 59 men and 166 women Duration of suffering from the illness: 9.8 ± 6.7 years 70.7% of the caregivers were married and 45.3% had a high school diploma. Most of the patients (32.4%) were 40–50 years old, and most of the caregivers (28.4%) were 50–60 years old,
Mashayekhi [46*]	2014	175	Thalassemia	ZARIT	49.88	Mean age of the caregivers: 38.1 ± 9.3 years Mean age of the patients: 10.7 ± 4.8 years All of the caregivers were mothers of patients.
Garmabi [47*]	2014	123	Psychology	CBI	75.59	Demographic characteristics of patients and caregivers not reported.
Salmani [19*]	2014	60	Cancer	CBI	84.82	Mean age of the patients: 38.4 ± 9 years Mean age of the caregivers: 43.6 ± 19.6 years Duration of suffering from the illness: 17.7 ± 16.2 months Gender of the caregivers: 49 women and 11 men Most of the caregivers (50%) were children of patients.
Abbasi [48*]	2013	133	Cancer	CBI	57.60	Gender of the caregivers: 67 women and 66 men Mean age of the caregivers: 35.7 ± 14.3 years Duration of suffering from the illness: 16.5 ± 19.5 months Most of the caregivers (51.9%) were children of patients. Most of the patients (28.6%) had breast cancer. Most of the caregivers (62.4%) were married.
Abdollahpour [49*]	2012	153	Psychology	CBI	47.58	Gender of the patients: 90 women and 63 men Mean age of the patients: 77.1 ± 7.4 years Mean age of the caregivers: 53 ± 13 years 88 of the caregivers were children of patients (69 were daughters and 19 were sons of patients).
Haresabadi [50*]	2012	75	Psychology	ZARIT	71.13	Mean age of the patients: 34.8 ± 12.2 years Gender of the patients: 43 men and 32 women Duration of suffering from the illness: 6.2 ± 6.3 years Mean age of the caregivers: 40.1 ± 12.2 years Gender of the caregivers: 35 men and 40 women
Navidian [51*]	2008	125	Psychology	ZARIT	47.9	Gender of the patients: 83 men and 42 women 59.2% of the patients were 20–35 years old. Gender of the caregivers: 59 men and 66 women 52% of the caregivers were 20–35 years old.
Mohammadi Shahbalagy [29*]	2006	81	Alzheimer’s disease	MCSI	50.82	Gender of the caregivers: 49 women and 32 men 56% of the caregivers were wives or husbands of patients.

CBI: Caregiver Burden Inventory; MCSI: Modified Caregiver Strain Index

**Fig. 2:**
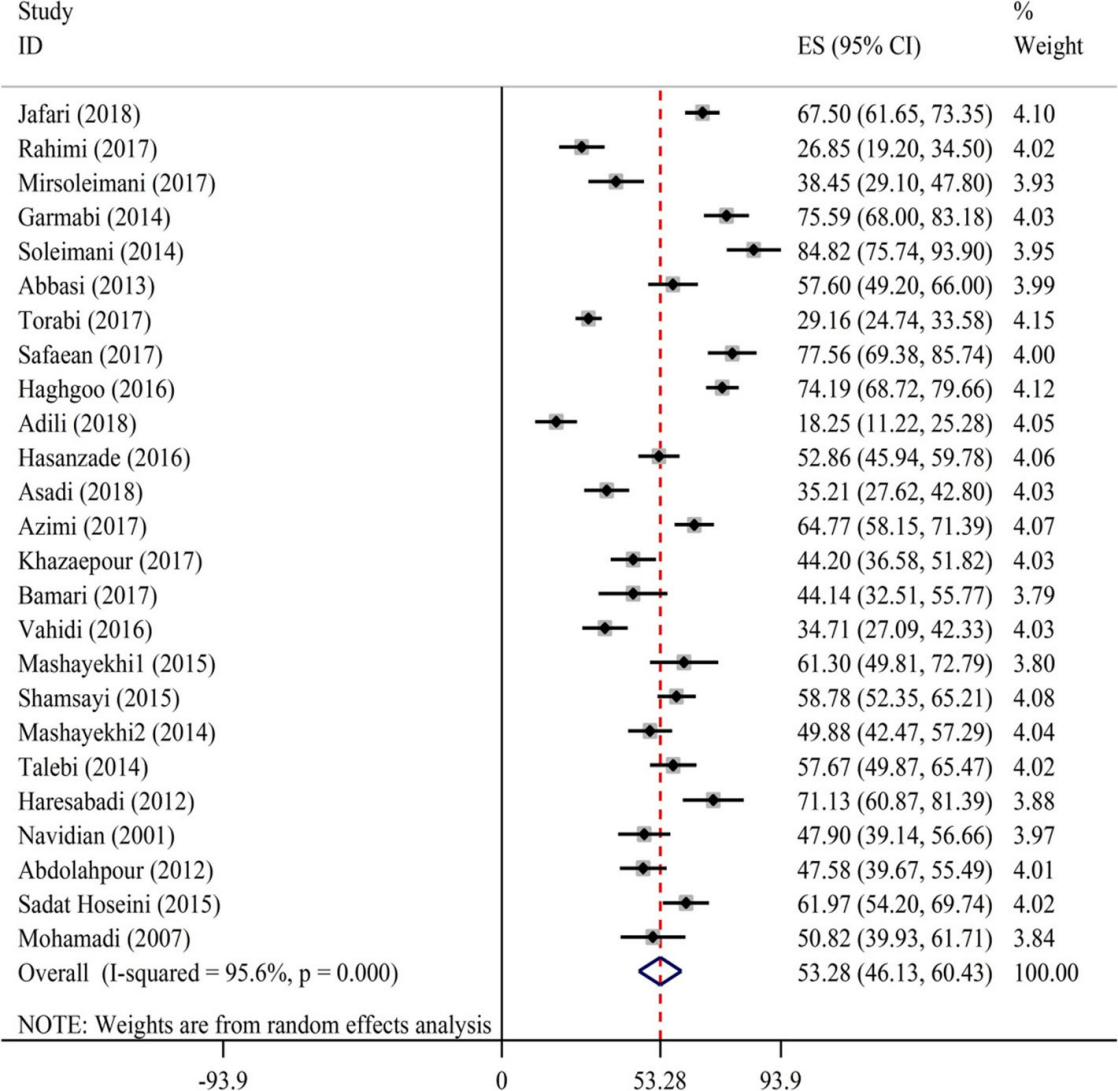
Burden-of-care scores for chronic patients based on the first author and year of study. The middle point in each line represents the standardized score for Iranian chronic patients; also, the diamond shows the overall score for all studies

All of the selected articles had average methodological quality. In the study, 25 articles with a total sample size of 3806 (on average, 152 participants per study) were reviewed systematically. The percentage of burden of care in caregivers of chronic patients was found to be 53.28% (95% CI: 46.13–60.43). The highest (84.82) and lowest (18.25) burden of care scores had been reported by Salmani [19*] and Adili [33*], respectively (Fig. 2).

The highest burden of care scores (%) belonged to caregivers of patients undergoing dialysis (62.75; 95% CI: 56.11–69.38), patients with mental disorders (58.69; 95% CI: 49.7–67.69), and patients with Alzheimer’s disease (57.07; 95% CI: 46.23–67.92). On the other hand, the lowest burden of care score (%) was associated with diabetes (34.92; 95% CI: 18.01–51.82).

Result of subgroup analysis by instrument (the CBI, the ZARIT etc.) showed that the highest burden of care was reported in studies using the CBI (54.96; 95% CI: 39.52–70.39). The highest and the lowest burden of care were related to the provinces located in regions 2 and 3, respectively. More details on burden of care scores (%) for different disorders, geographical areas, and tools are provided in Table 3.

**Table 3:** Burden-of-care scores for different disorder subgroups, areas and tools

Groups		Number of Studies	Sample Size	Score (%)	95% CI	Heterogeneity	
						I2	P
Type of disease	Hemodialysis	3	469	62.75	56.11–69.38	51	0.130
	Diabetes	2	199	34.99	18.01–51.82	83.1	0.015
	Cancer	6	663	51.84	30.55–73.13	97.5	0.001
	Mental Disorder	9	1499	58.69	49.70–67.69	92.8	0.001
	Alzheimer’s	2	231	57.07	46.23–67.92	62.5	0.102
	Others	3	745	40.81	27.23–54.40	92.6	0.001
Country Areas	1	10	1423	51.23	40.76–61.70	94.4	0.001
	2	1	246	74.19	68.72–79.66	–	–
	3	3	711	40.31	23.72–56.91	94.9	0.001
	4	4	753	47.21	28.44–65.97	94.9	0.001
	5	7	674	62.47	50.43–74.51	91.5	0.001
Tool	CBI	10	1664	54.96	39.52–70.39	98	0.001
	Zarit	11	1558	51.69	44.67–58.70	87.7	0.001
	Others	4	584	53.50	47.25–59.76	57	0.073

Area 1: Provinces of Tehran, Alborz, Qazvin, Mazandaran, Semnan, Golestan, and Qom; Area 2: Provinces of Esfahan, Fars, Boushehr, Chaharmahal Bakhtiari, Hormozgan, and Kohkilouye and Boyer Ahmad; Area 3: Provinces of Azarbayjan Sharqi, Azarbayjan Qarbi, Ardebil, Zanjan, Gilan, and Kordestan; Area 4: Provinces of Kermanshah, Ilam, Lorestan, Hamedan, Markazi, and Khouzestan; Area 5: Provinces of Khorasan Razavi, Khorasan Jonoubi, Khorasan Shomali, Kerman, Yazd, and Sistan and Balouchestan

**Fig. 3:**
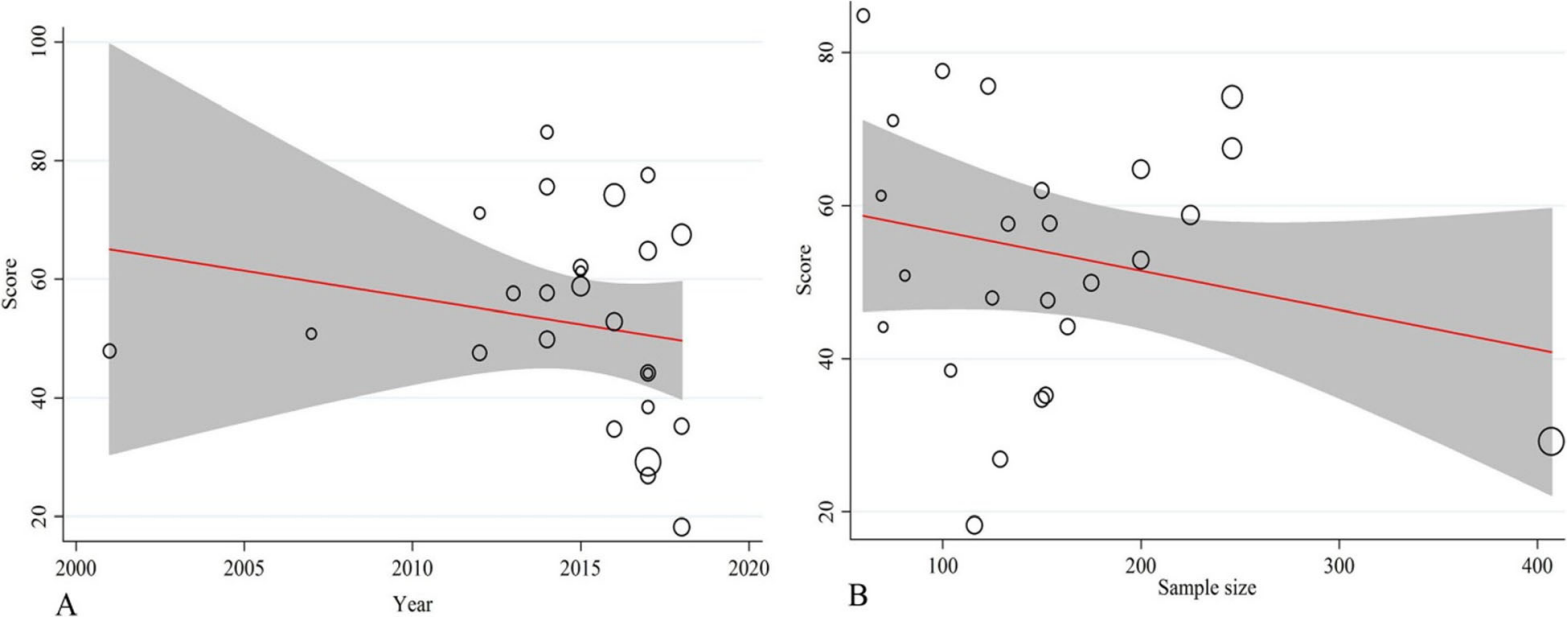
Meta-regression of burden-of-care score (%) versus year (A) and sample size (B). The circles show the weights of the studies

**Fig. 4:**
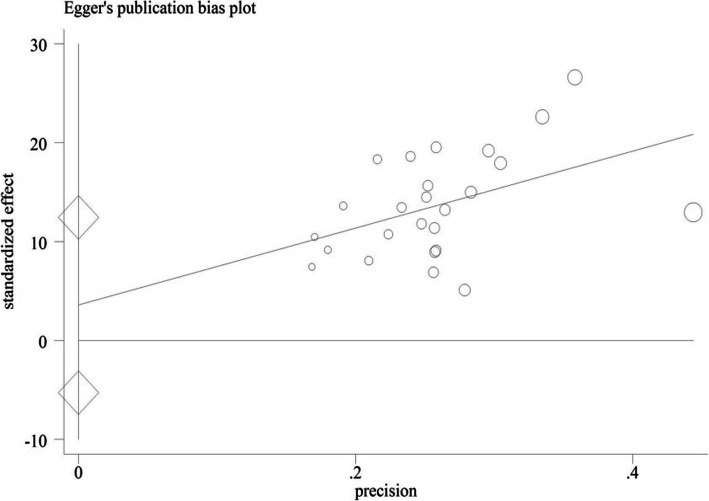
Publication bias

As shown in Fig. 3, the meta-regression results indicated no significant correlation between the mean burden of care score and year of publication (*p* = 0.507) and sample size (*p* = 0.407) (Fig. 3). Sensitivity analysis showed that none of the studies alone had a significant effect on the overall estimation of the total percentage of burden of care. Moreover, as shown in Fig. 4, publication bias was not significant (*p* = 0.84) (Fig. 4).

## Discussions

The present study was aimed at estimating the overall percentage of burden of care in caregivers of Iranian patients with chronic disorders. The burden of care scores indicated that the caregivers experienced high levels of burden of care. The findings of this research are in line with those reported in the international literature. For example, Etters et al. reported a high burden of care in caregivers of patients with mental disorders [55]. Also, Bayoumi et al. reported a higher-than-average burden of care in caregivers of dialysis patients (20.1%) [56]. These results agree with the current research. In the present study, the highest level of burden of care was observed in caregivers of hemodialysis patients. Consistent with this finding, Mollaoğlu showed that caring for a hemodialysis patient is very stressful and has an adverse impact on physical, psychological, and mental wellbeing of caregivers (59.2%) [57].

The adverse health effects of Uremia affect all organs of the body and lead to impairments and lowered quality of life. The impact of dialysis on the life of patients and their families is so deep that may lead to adjustment problems, and inevitably increase the burden of the care for the caregiver [58]. Caregivers of patients with mental disorders face issues, such as interpersonal problems, role conflicts, stress, and constant anxiety in life, thereby imposing a high burden of care on the caregiver. In fact, due to the high level of impairment the patient experiences, the caregiver may feel more responsible to take care of the patient, therefore experiencing a higher level of stress and tension. This is in line with the findings of Steele and Covinsky [59, 60]. Due to the stigma surrounding seeking professional help for mental disorders in Iran, most caregivers may not be willing to talk about their mental problems. Therefore, this issue is expected to be more common than what is recorded [61, 62]. The high level of burden of care in caregivers of patients with Alzheimer’s disease can be related to deep dependence of these patients to their caregivers, which leads to different problems in the long term. The burden of care experienced by caregivers of these patients is so high that some researches consider the caregivers as hidden patients [63–65]. Patients with Alzheimer’s disease are often old and their dependence and inability to care for themselves may lead to mental disorders in their caregivers. The symptoms of depression among these caregivers are reported to be as twice as in other caregivers [65]. In Takai’s study, caregivers of patients with Alzheimer’s believed that caregiving had decreased their health and had made them vulnerable to fatigue and mental disorders [66].

In this research, patients with diabetes had the lowest burden of care score (%). In line with this finding, in Kim’s study that was focused on examining burden of care in caregivers of patients with cancer, mental disorders, Alzheimer’s disease, diabetes, and other disorders, a higher burden of care was reported for patients with cancer and dementia compared to those with diabetes [67]. Based on previous studies conducted among patients with chronic disorders, those with diabetes have an average quality of life. It has been shown that patients with chronic disorders can become less dependent on their caregivers through self-care activities and controlling the symptoms of their illness [68, 69]. This can reduce the burden of care faced by the caregiver.

The results of meta-regression analysis showed that between 2007 and 2018, there has been no change in the overall burden of care score (%) of caregivers of Iranian patients with chronic disorders, although a decrease was expected in the score given the emergence of new methods of care. It appears that scientific methods are not implemented properly in many treatment centers across Iran, and caregivers of the patients may not receive adequate training. Therefore, caregivers bear a lot of pressure that may undermine their caregiving abilities over time. As a result of this, patients may need to seek help from treatment centers. One of the strengths of the present study is that for the first time, it has investigated and compared burden of care for caregivers of patients with chronic disorders in Iran. One of the limitations of the study was that grey literature (e.g. working papers, research reports, conference proceedings) was not included in the analysis, because there was no comprehensive database for grey literature in Iran. Another limitation was that some details, such as raw score of burden of care by gender had not been reported in the selected studies; this prevented further examination.

## Conclusion

Caregivers of Iranian patients with Chronic disorders, especially those caring for patients undergoing dialysis, patients with mental disorders, and patients with Alzheimer’s disease experience high levels of burden of care. Therefore, necessary measures need to be taken by Iranian health care officials to reduce burden of care in this group.

## Abbreviations

CBI: Caregiver Burden Inventory
CI: Confidence Interval
MCSI: Modified Caregiver Strain Index
PRISMA: Preferred Reporting Items for Systematic Reviews and Meta-Analyses
SID: Scientific Information Database
STROBE: Strengthening The Reporting of OBservational Studies in Epidemiology
ZBI: Zarit Caregiver Burden Interview
